# Conditional knock out of transcription factor CTCF in excitatory neurons induces cognitive deficiency

**DOI:** 10.1186/s13041-020-00716-z

**Published:** 2021-01-05

**Authors:** Dong Il Choi, Myeongwon Kim, Somi Kim, Nam-Kyung Yu, Chuljung Kwak, Hyunhyo Seo, Kyungmin Lee, Bong-Kiun Kaang

**Affiliations:** 1 School of Biological Sciences, College of Natural Sciences, Seoul National University, 1 Gwanangno, Gwanak-gu, Seoul, 08826 South Korea; 2 Laboratory for Behavioral Neural Circuitry and Physiology, Department of Anatomy, Brain Science and Engineering Institute, School of Medicine, Kyungpook National University, 680 Gukchaebosang-ro, Jung-gu, Daegu, 41944 South Korea

## Abstract

CCCTC-binding factor (CTCF) is a transcription factor that is involved in organizing chromatin structure. A reduction of CTCF expression is known to develop distinct clinical features. Furthermore, conditional knock out (cKO) study revealed reactive gliosis of astrocytes and microglia followed by age-dependent cell death in the excitatory neurons of *CTCF* cKO mice. To assess the cognitive ability in *CTCF* cKO mice of over 20 weeks of age, we examined pairwise discrimination (PD), PD reversal learning (PDr), and different paired-associate learning (dPAL) tasks using a touch screen apparatus. We found cognitive impairment in dPAL touch screen tests, suggesting that prolonged *Ctcf* gene deficiency results in cognitive deficits.

CTCF is a highly conserved zinc finger nuclear protein [1], acting as a genome organizer and a transcription factor. It is multifunctional organizer involved in chromatin remodeling by binding to nuclear genome through its zinc finger motifs to recruit other transcriptional regulators, to insulate the promoters and enhancers in the genome [1, 2]. Given this critical genome-wide function, CTCF knockout mice are embryonic lethal. Therefore, conditional knockout approaches have been used to examine its role in the brain [3–5]. We showed that *Ctcf* gene deficiency leads to altered gene expression and impaired synaptic plasticity in the forebrain of adult mice [4, 6]. Moreover, recently we also showed neuroinflammation with reactive gliosis in the cortex from 16 weeks of age accompanied by neuronal cell death at over 20 weeks of age in forebrain glutamatergic neuron-specific *Ctcf* gene KO (*CTCF* cKO) mice [7].

*CTCF* cKO mice have been previously reported to exhibit impairments to hippocampus-dependent memory and cortex-dependent remote memory [4, 5]. However, these studies were performed in relatively young *CTCF* cKO mice (10–20 weeks old). Given the neurodegeneration-like changes in the cortex of cKO mice, we wanted to examine cognitive impairment of prolonged *Ctcf* gene deficiency in animals > 20 weeks old using a more complex behavioral paradigm by applying the mouse touch screen test [8]. As touch screen tests are also used for humans, they are relatively more complex and difficult than most mouse battery tests. Touch screen tests require weeks of pre-training before the actual test, hence providing reliable data on mouse learning skills and test results.

The touch screen-based behavioral experiments for pairwise discrimination (PD), PD reversal learning (PDr) and different paired-associate learning (dPAL) tasks were performed as described in our previous studies [9, 10] (Additional file 1). When mice fulfilled the success criteria (correct response > 80%) in PD learning, we performed PD reversal learning by switching S+ (reward giving stimulus) and S− (beep alert stimulus) to measure behavioral flexibility. We examined the learning performance over time in control and cKO mice for 10 days (Fig. 1a) with linear regression analysis (Fig. 1b). We found that the learning enhancement per day (%) is significantly lower in cKO group during reversal leaning compared to control group (Fig. 1c; control = 7.4496 ± 0.713%/day, cKO = 3.3246 ± 1.124%/day; t(11) = 2.973, *p = 0.012668; independent *t* test). These data suggest that the ability to suppress a previous reward-related response is decreased in cKO, indicating a deficit of behavioral flexibility in cKO mice.

**Fig. 1:**
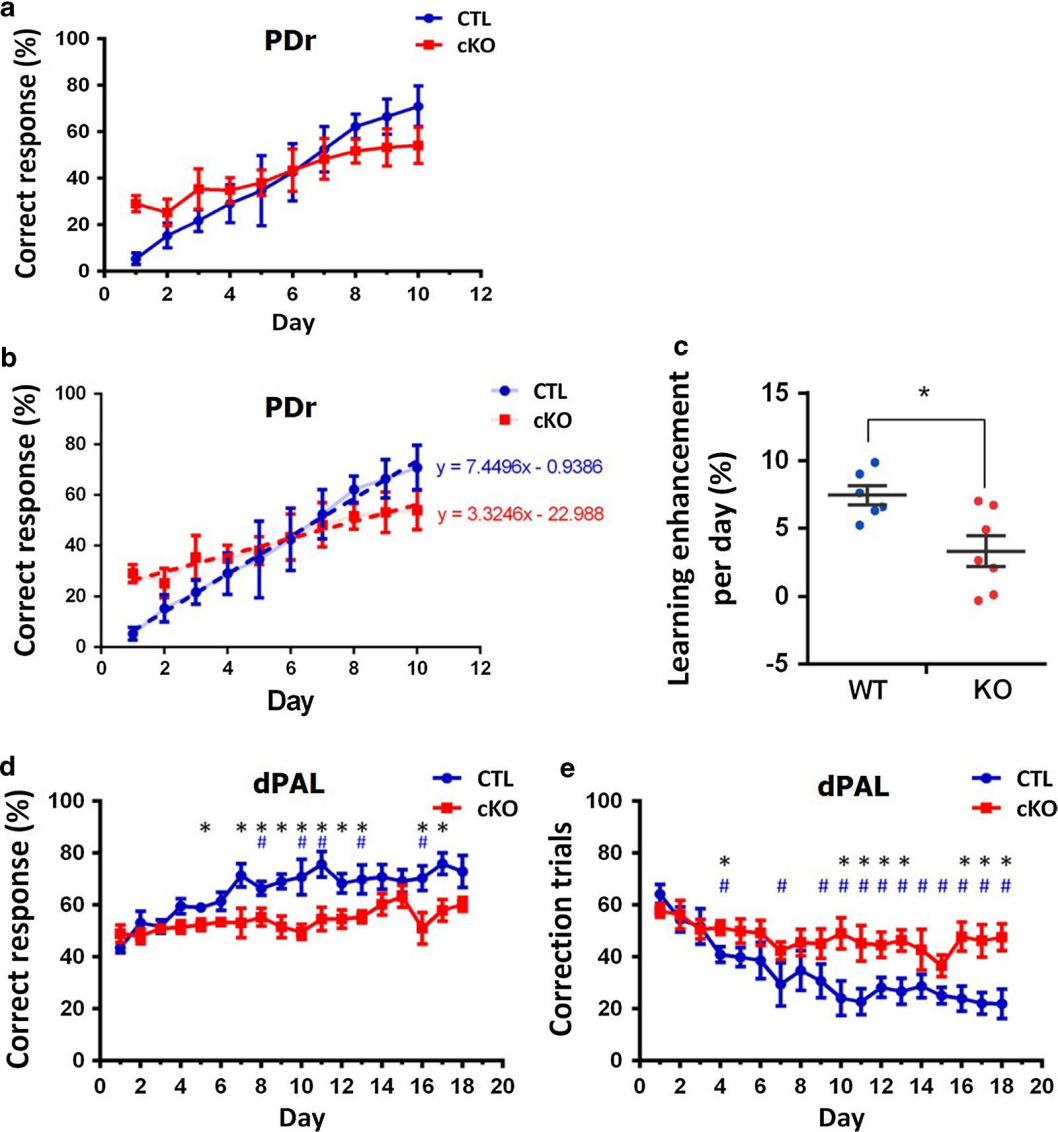
Touch screen analysis of behavioral phenotypes showed cognitive impairments in *CTCF* cKO mice. **a** PD reversal accuracy (i.e., correct response) rate. **b** Linear regression between correct response rate and time in the PD reversal leaning. **c** Comparison of reversal learning rate per day (%) between control and cKO mice. Individual dots represent data from individual animals. **d** Accuracy rate of different paired-associate learning (dPAL) touch screen test trials was measured after habituation and pre-training. In the dPAL test, adult *CTCF* cKO mice showed a moderate cognitive impairment. *p < 0.05, ^*#*^*p* < 0.05. **e** Time course of learning phase during dPAL test. Note that the number of correction trial is decreased more rapidly in the controls than in cKO mice which showed no difference in the number of correction trial from day 1 to day 18. *p < 0.05, ^*#*^*p* < 0.05

Furthermore, in the dPAL test, compared to the controls, *CTCF* cKO mice showed impairments in selecting the correct response. Although *CTCF* cKO and control mice started the training with a similar level of correct responses, *CTCF* cKO mice displayed a learning deficit as training proceeded and did not reach the same level of correct responses as control mice (Fig. 1d). In the dPAL data analysis, we found a significant interaction between “genotype” and “time” (Fig. 1d; genotype: F(1, 11) = 12.551, *p* = 0.005, time: F(9.809, 107.898) = 6.513, *p* = 0.0000001, interaction: F(9.809, 107.898) = 2.459, *p* = 0.011; two-way ANOVA with mixed design repeated measures). Simple main effect analysis as a post-hoc revealed that *CTCF* cKO mice showed impairments in selecting the correct response compared to the controls (Fig. 1d; for day 5, *p = 0.037; for day 7, *p = 0.031; for day 8, *p = 0.030; for day 9, *p = 0.006; for day 10, *p = 0.013, for day 11, *p = 0.009, for day 12, *p = 0.021, for day 13, *p = 0.027, for day 16, *p = 0.034, for day 17, *p = 0.012). Although *CTCF* cKO and control mice started the training with a similar level of correct responses at day 1, *CTCF* cKO mice displayed a moderate learning deficit as training proceeded and did not reach the same level of correct responses compared with the control mice showing an enhancement of correct response over time (Fig. 1d; for day 8, ^*#*^*p* = 0.040; for day 10, ^*#*^*p* = 0.035; for day 11, ^*#*^*p* = 0.033; for day 13, ^*#*^*p* = 0.037; for day 16 ^*#*^*p* = 0.010; simple main effect analysis with pairwise comparison). During the training periods, the number of correction trials was analyzed in *CTCF* cKO and control mice by two-way ANOVA with repeated measures and we found a significant interaction between “genotype” and “time” (mixed model ANOVA; Fig. 1e; genotype: F(1, 11) = 6.304, *p* = 0.029, time: F(9.814, 107.950) = 9.571, *p* = 0.000000000039, interaction: F(9.814, 107.950) = 2.958, *p* = 0.003). Then, simple main effect analysis as a post-hoc revealed that the control mice showed a significant decrease in the number of correction trials over time compared to cKO mice (Fig. 1e; for day 4, *p = 0.027; for day 10, *p = 0.017; for day 11, *p = 0.026; for day 12, *p = 0.025; for day 13, *p = 0.011, for day 16, *p = 0.009, for day 17, *p = 0.010, for day 18, *p = 0.007). Moreover, when comparing the correction trial number of day 1 with each one of day 2 to day 18, the number of correction trials decreased more rapidly in the controls than in cKO mice showing no difference over time (Fig. 1e; in control mice: for day 4, ^*#*^*p* = 0.031; for day 7, ^*#*^*p* = 0.031; for day 9, ^*#*^*p* = 0.049; for day 10, ^*#*^*p* = 0.005; for day 11, ^*#*^*p* = 0.003; for day 12, ^*#*^*p* = 0.001; for day 13, ^*#*^*p* = 0.002; for day 14, ^*#*^*p* = 0.045; for day 15, ^*#*^*p* = 0.002; for day 16, ^*#*^*p* = 0.003; for day 17, ^*#*^*p* = 0.004; for day 18, ^*#*^*p* = 0.002; simple main effect analysis with pairwise comparison).Taken together, these results indicate that *CTCF* cKO mice show a deficit in behavioral flexibility and cognitive function, which may be related to the cell death and reactive gliosis caused by CTCF deficiency.

In this study, we showed that adult *CTCF* cKO mice exhibited signs of lack of behavioral flexibility and cognitive dysfunction in the PDr and dPAL touch screen test. These cognitive impairments are likely due to the loss of glutamatergic neurons in the cortex, particularly in the ACC, as inhibitory neurons in the striatum are relatively intact in adult *CTCF* cKO mice [7]. ACC is known to be involved in behavioral flexibility as well as decision making, adaptation, and anticipation in rodents [11]. Therefore, it is possible that CTCF deficiency in the ACC may lead to lack of behavior flexibility in adult *CTCF* cKO mouse. Although CTCF deficiency has been demonstrated to be prominent in the ACC [7], which plays a critical role in remote memory formation [12, 13], pain processing [12], and other higher cognitive functions [14], other forebrain areas may also contribute to the cognitive dysfunction induced by CTCF deficiency. As we reported previously, the hippocampus plays a key role in dPAL leaning [9]. Interestingly, it was reported that direct glutamatergic projection from the ACC to hippocampal CA1 and CA3 region is involved in retrieval of contextual fear memory [15]. In this regard, loss of glutamatergic neurons in the ACC of *CTCF* cKO mice and the hippocampus may affect retrieval of association between location and visual stimuli during dPAL learning. Further, although 12–15-week old *Ctcf* cKO mice did not show any deficit in learning and recent memory storage during contextual fear conditioning and Morris water maze task [5], it is still possible that younger mice at earlier ages could show a deficit in our touch screen-based cognition test that remains to be resolved in the future.

## Supplementary Information

**Additional file 1.** Supplementary materials & methods.

## Data Availability

The data supporting the conclusions of this study are included in this article and Additional file 1. Additional data are available from the corresponding author upon request.
