# Supplementary material for: Conditional knock out of transcription factor CTCF in excitatory neurons induces cognitive deficiency
Source: Mol Brain. 2021 Jan 5;14:1. doi: 10.1186/s13041-020-00716-z (PMC7784033; doi:10.1186/s13041-020-00716-z)
Supplement: Supplementary file 1 — Additional file 1. Supplementary materials & methods. [file 13041_2020_716_MOESM1_ESM.docx]

**ADDITIONAL MATERIALS**

**Animals**

We generated *CTCF* cKO mice as described in Kwak et al. [7] by crossing *CTCFfl/+;CaMKIIα+/+* with *CTCFfl/+;CaMKIIαCre/+*. We used offsprings genotyped with *CTCFfl/fl;CaMKIIαCre/+* as the *CTCF* cKO mice group. We used as controls littermates without the floxed *CTCF* or the *Cre* transgene. Thirty-week-old male mice were used for behavioral analyses. All animals were housed under a 12-h light/dark cycle with water and food provided *ad libitum*. The Animal Care and Use Committees of Seoul National University approved the animal protocols.

**Touch screen behavioral tests**

The touch screen protocol consisted on habituation, a series of pre-training, PD, PDr, and dPAL task. Mice were screened through a pre-training process; subjects that observed the criteria performed the experiment. Pre-training session consists of a series of trainings*:* initial touch training, must touch training, and incorrect punishment. After completion of pre-training session, the mouse went on to PD, PDr and dPAL tasks.

***Habituation***
Habituation was done as described before [9, 10]. After food restriction initiation, mice were handled for 2 days. During a 10-min session, the mice were habituated to the touchscreen chambers without any signals for 1 day. On the next day, the mice were habituated to the operant chambers with access to the liquid reward, which was administered every time the mouse’s nose poked the illuminated reward magazine for 40 min.

***Pre-training***
Touchscreen pre-training was conducted as described before [9, 10] in order to progressively train mice to touch the visual stimuli on the screen and receive their reward.

*Initial touch training*: mice obtained the liquid reward by touching various shapes appearing on one of the three screen windows.
*Must touch training*: a trial began when the mouse’s nose poked the illuminated reward magazine. Then, a square was displayed on one window, and the liquid reward was released only when the mice touched the stimulus.
*Incorrect punishment*: a punishment was added to the must touch training. If the mice touched a window with no stimulus (blank), a time-out was given for 5 s. When mice gave a correct response during the correction trial, another normal trial was administered. When each mouse completed 30 trials or 60 min had elapsed, a session was considered finished. Furthermore, when a mouse attained 75% accuracy for 2 out of 3 days, the mouse went on to PD, PDr and dPAL tasks.

***PD and PDr tasks***

In PD, there were two distinguishable choices in the screen: a triangle shape S+ and a star shape S-. When an animal touched the S+ stimulus, it received a liquid reward. But when choosing S-, it received a beep alert instead of a reward. The positions of S+ and S- on the screen were chosen at random in each trial. We performed 30 trials in an hour. We continued training for 6 to 12 days until we got 80% correct responses on 3 out of 4 days. The entire period of PD training was not statistically different between two genotypes (control 7.5 ± 0.5 days (n = 6); cKO, 8.7 ± 0.9 days (n = 7); t(11) = -1.105, *p* = 0.293, independent *t* test). Then we performed PDr by switching the outcomes of S+ and S- to measure behavioral flexibility under the same criterion.

***dPAL task***

dPAL was basically done as described before [9, 10]. A trial started when the mouse poked its nose into the illuminated magazine. During each task, six different combinations of stimuli were presented: a flower on the left window, an airplane on the middle, and a spider on the right, all of which were regarded as correct stimuli. When mice touched the correct stimuli, they received the liquid reward and an inter-stimulus interval (ITI) (15 s) was introduced. However, when the nose poked an incorrect stimulus, a time-out (5 s) with low buzzer tone and an ITI was given before the next trial began. A session ended when a mouse completed 60 trials or 60 min had elapsed. In the correction trial, when the mice select the wrong stimulus, a time-out is given for 5 seconds. After that, the same stimulus arranged as before is given to the mice, in order to give a chance to learn the correct answer. If the mouse continues to select the wrong answer, the correction trial will be repeated and the number of correction trials would increase. In this case, even though the mouse makes the right choice in the correction trial stage, the correct percentage, which is the percentage of correct answers, does not increase.

**Experimental Design and Statistical Analysis**

All experiments were carried out in a blinded fashion. We used eight to sixteen male mice for behavioral tests to produce statistical validity. The behavioral data were analyzed by two-way ANOVA with mixed design repeated measures, where genotype as a between-subject factor and time as a within-subject factor. Huynh-Feldt corrected F statistics and degree of freedoms were used when sphericity was violated. Simple main effect with pairwise comparison was analyzed as post-hoc when the result of repeated measures ANOVA shows significant interactions between factors. For analysis of learning performance over time in PD reversal learning, linear regression was conducted to model the relationship between correct response rate and time. The linear regression slope means a rate of learning enhancement per day and the significance of difference in linear regression slope between control and cKO mice was confirmed by independent *t* test. Data are shown as mean ± standard error of the mean.
